# Parental anxiety in China's social media marketing of early orthodontics

**DOI:** 10.3389/fpubh.2026.1814711

**Published:** 2026-05-12

**Authors:** Xing Wang, Wenfeng Li, Xin He

**Affiliations:** 1 Department of Stomatology, China Rehabilitation Research Center, Beijing, China; 2 Department of Stomatology, PLA Rocket Force characteristic Medical Center, Beijing, China

**Keywords:** social media, anxiety, early orthodontics, paediatric dentistry, health China 2030

## 1 Introduction

The expansion of China's early orthodontic market is increasingly driven by parental anxiety. This emotion can be deliberately created and used for profit in today's digital world. Social media has become a primary health information source for Chinese adults. A national survey reported that 71.9% obtain health education online (1). Yet misleading information is widespread on these platforms (2), and users often struggle to evaluate health content (3).

Between 2018 and 2022, orthodontic related content on Chinese social media grew roughly four-fold. Orthodontics surpassed general dentistry as the fastest-growing oral health topic (4). Yet the quality of this information is deeply concerning. According to the same study, 79.4% of long-text dental posts were written by nonprofessionals. Using Spearman correlation analysis, the researchers found a significant negative correlation emerged between user engagement and content quality. On Weibo the correlation coefficient was −0.45 (*P* = 0.01). On WeChat it was −0.30 (*P* = 0.02). The most widely viewed content is often the least reliable (4). A separate analysis of pediatric orthodontic videos on TikTok confirmed this pattern. Orthodontist produced content scored significantly higher on reliability metrics with a P value below 0.01. Yet algorithm driven engagement consistently favored lower-quality material. The most viewed videos offered limited guidance on treatment timing or indications (5).

Importantly, the gap between parental concern and clinical necessity is not a new phenomenon. Early European studies found that parents' perceptions of their children's dentition often diverge from orthodontic expert assessments. Parental anxiety can exceed clinically indicated needs (6, 7). These findings suggest that parental worry can operate independently of objective disease burden. In today's digital world, this vulnerability is amplified.

Parental anxiety does not arise naturally. It is amplified within a digital environment where low-quality content receives the most attention. This trend risks undermining evidence-based care and may widen health inequalities.

## 2 Commercialization, digital marketing, and the exploitation of parental anxiety

Malocclusion affects 45.50% of Chinese preschool children, a condition that without appropriate intervention can lead to severe dentofacial deformities (8). Pediatric malocclusion management is uniquely complex, shaped by individual craniofacial growth patterns (9). China has developed clinical consensus documents for early orthodontic intervention, including an expert consensus published in 2021 (10) and subsequent guidelines for specific conditions (11). However, a gap exists between these evidence-based recommendations and clinical practice. A recent analysis notes that some clinicians continue to perform excessive or premature interventions and exaggerate the role of early orthodontic treatment (12). This gap can create uncertainty for parents seeking guidance.

Since the 2009 healthcare reforms, China's private dental sector has expanded to over 130,000 clinics nationwide, far outnumbering public facilities (13). While public hospitals, particularly tertiary stomatology hospitals, continue to handle a substantial proportion of patient volume, private clinics have come to dominate the direct-to-consumer marketing landscape. Facing intense competition and high customer acquisition costs (14), these clinics increasingly rely on social media platforms to attract patients. Recently, orthodontic topics have represented the fastest-growing category of oral health discussion, with a substantial proportion of high-engagement posts containing promotional material (4).

Pediatric orthodontics, a high-margin service, has attracted considerable commercial attention in this digital space. Content often includes case demonstrations, warnings about risks of delayed treatment, and claims about optimal timing for intervention (4). Such promotional practices have contributed to misconceptions about early orthodontic care, including exaggerated claims and premature interventions (12). Unlike adult orthodontics, where treatment goals are relatively stable, pediatric intervention requires careful consideration of individual craniofacial growth patterns (9). Treatment decisions must balance the potential benefits of early intervention against the risks of unnecessary treatment. This complexity makes the specialty particularly vulnerable to inappropriate early intervention when commercial incentives override clinical judgment.

A related concern is the potential blurring of professional boundaries in digital health communication. When clinicians use their institutional affiliations in commercial social media content, parents who trust the impartiality of public hospitals may find it hard to tell education from promotion. This issue has drawn regulatory attention, in part because hospital social media activity has grown substantially. A cross-sectional study of 721 officially verified hospital Weibo accounts found that promotional strategies significantly influence audience reach and engagement. The study identified hospital ownership, whether public or private, as a key factor shaping the effectiveness of social media promotional strategies (15). This suggests that public hospitals may face distinct challenges or incentives in managing their digital presence.

## 3 Regulatory responses and remaining challenges

The regulatory landscape has responded to these challenges. Since 2022, TikTok has prohibited medical professionals from engaging in live commerce. The Chinese National Health Commission has also announced strict monitoring of public hospital doctors' participation in pharmaceutical advertising and disguised medical promotions (16). Earlier position statements from professional associations noted the need to oppose excessive commercialization in dental practice (17).

These patterns of commercial promotion raise questions about equity in pediatric dental care. Private clinics are predominantly concentrated in economically advanced urban areas (13). This means families with greater financial means are more likely to encounter such marketing. Access to evidence-based pediatric dental services is uneven across socioeconomic groups. A national study found that dental care utilization among preschool children was concentrated in higher socioeconomic status families, with a concentration index of 0.103. Household income accounted for 45.66% of this inequality, while maternal education explained another 24.22% (18). This dual dynamic presents a challenge to the equity principles articulated in the Healthy China 2030 framework.

The 2025 Medical Advertising Identification Guide, jointly issued by three national authorities, now explicitly classifies covert promotional tactics such as unlabelled science popularization and patient testimonials as unlawful medical advertising (19). This represents an important step forward. However, challenges in implementation remain.

First, distinguishing genuine education from disguised advertising continues to rely on case-by-case judgment. The official interpretation of the guidelines acknowledges that medical advertising, medical information, and health science popularization are often similar in form. This makes enforcement subjective in practice. Second, platform accountability mechanisms are still evolving. The 2025 Guide includes provisions requiring internet platforms to verify the qualifications of medical content producers. Whether these requirements can effectively curb algorithmic amplification of misleading content requires further evaluation. Third, commercial activity by public hospital clinicians remains an area of regulatory focus. Recent studies show that a substantial number of doctors from tertiary hospitals maintain verified social media accounts. Professional associations have also issued statements opposing excessive commercialization in dental practice (16, 17). Fourth, investment in parental health literacy remains a gap. This matters because parents who lack adequate skills to evaluate health information may experience confusion and anxiety when they encounter conflicting content online (20).

## 4 Discussion

This phenomenon reflects broader tensions in China's healthcare system. As the private sector grows alongside public services, it becomes harder to balance commercial interests with public health goals. Social media algorithms are built to keep users engaged. Research shows that certain message features, such as emotional appeals and narrative style, can increase people's intention to share health information, even when that information is misleading (21). When parents encounter conflicting health information online, they may feel confused and anxious. These dynamics can make them more vulnerable to commercial promotion.

Addressing this requires practical steps. Professional associations can update and share clear clinical guidelines for early orthodontic treatment. Policymakers can expand public dental services in areas that need them most. These efforts should follow the Healthy China 2030 goal of fair access to care. For dentistry to serve the public good, commercial practices must support this mission rather than work against it.
